# Enhancing vestibular function in the elderly with imperceptible electrical stimulation

**DOI:** 10.1038/s41598-017-18653-8

**Published:** 2018-01-10

**Authors:** Jorge M. Serrador, Brian M. Deegan, Maria C. Geraghty, Scott J. Wood

**Affiliations:** 1 Rutgers Biomedical and Health Sciences, Depart. of Pharmacology, Physiology & Neuroscience, Newark, NJ USA; 2 National University of Ireland Galway, Galway, Ireland; 3 University College Dublin, Dublin, Ireland; 4 Azusa Pacific University, Dept. of Psychology, Azusa, CA USA; 5 NASA Johnson Space Center, Space Medicine Operations, Houston, TX USA; 6 War Related Illness & Injury Study Center, NJ Health Care System, Dept of Veteran Affairs, East Orange, NJ USA

## Abstract

Age-related loss of vestibular function can result in decrements in gaze stabilization and increased fall risk in the elderly. This study was designed to see if low levels of electrical stochastic noise applied transcutaneously to the vestibular system can improve a gaze stabilization reflex in young and elderly subject groups. Ocular counter-rolling (OCR) using a video-based technique was obtained in 16 subjects during low frequency passive roll tilts. Consistent with previous studies, there was a significant reduction in OCR gains in the elderly compared to the young group. Imperceptible stochastic noise significantly increased OCR in the elderly (Mean 23%, CI: 17–35%). Increases in OCR gain were greatest for those with lowest baseline gain and were negligible in those with normal gain. Since stimulation was effective at low levels undetectable to subjects, stochastic noise may provide a new treatment alternative to enhance vestibular function, specifically otolith-ocular reflexes, in the elderly or patient populations with reduced otolith-ocular function.

## Introduction

The risk of vestibular dysfunction increases as a function of age^1^. Age-related vestibular loss has been demonstrated in multiple vestibular pathways^2^ and has been associated with increased falls^3^. Understanding age-related loss in vestibular function will therefore help characterize those who may be at greater risk of fall injury. More importantly, clinical interventions that can enhance vestibular function in the elderly would be expected to improve activity levels leading to better outcome measures of daily living^4^.

Ocular-counterroll (OCR) is one measure that has been shown to be sensitive to age-related loss in vestibular function^5,6^. OCR gain is a gaze stabilizing mechanism involving ocular torsion in a compensatory direction to lateral roll-tilt of the head^7^. At low frequencies of motion, OCR gain is predominantly mediated by the otoliths^8^. A recent study has demonstrated video-based measurement of OCR has high diagnostic value to detect bilateral and unilateral otolith loss^9^. We previously demonstrated that reductions in OCR gain with aging were correlated with increased postural sway, suggesting OCR loss may also a useful predictor of fall risk^5^.

Based on these previous findings, we chose OCR gain as a marker of vestibular loss to evaluate a method of enhancing gain in the elderly using low levels of imperceptible electrical stimulation. Noise is generally thought to be detrimental to the detection and transmission of signals but, under certain conditions noise can enhance weaker signals in nonlinear systems through the phenomenon of stochastic resonance^10^. For example, while higher levels of electrical vestibular stimulation (e.g., ~5 mA) have been used to evoke eye movements^11^ or postural instability^12^, recent studies have been using low levels of “noisy” electrical stimulation to improve vestibular function^13^. We previously used low levels of electrical stochastic noise (SN) to enhance tactile perception in both young^14^ and older human subjects^15^. In this study, we extend this work to examine the effects of imperceptible electrical SN on otolith-ocular function in both young and elderly subjects.

Our study used a cross-sectional design to compare the effect of age using both young and older subject groups. We then used a repeated measures approach to examine the effects of the electrical SN on OCR in both groups. Since we expected the OCR gains to be lower in the elderly, we hypothesized this group would show greater improvements with the SN intervention.

## Results

### Effects of aging on OCR

For both young and elderly groups, the OCR gain increased with frequency (P = 0.03, Table 1). This gain increase can be attributed to increasing canal input due to both higher frequencies of tilt and increased tilt velocity. Across all frequencies, the baseline OCR gains were significantly reduced for the elderly compared to the young group (P = 0.005). The lower OCR gains in this elderly group are in accordance with previous findings from our laboratory (N > 150)^5^. This difference between young and elderly was greatest at 0.03125 Hz at which the OCR gain was reduced by 57% versus 38% at the other two frequencies.

**Table 1:** Mean OCR gain values (±SEM) for young and elderly participants in baseline and SN trials at three frequencies of roll tilt.

Conditions	0.03125 Hz	0.125 Hz	0.2 Hz
Young Baseline	0.196 ± 0.020	0.221 ± 0.028	0.224 ± 0.027
Young SN	0.202 ± 0.019	0.243 ± 0.031	0.217 ± 0.033
Elderly Baseline	0.085 ± 0.018	0.137 ± 0.022	0.139 ± 0.021
Elderly SN	0.108 ± 0.017	0.158 ± 0.027	0.173 ± 0.028

### Enhancement of OCR using SN

**Figure 1:**
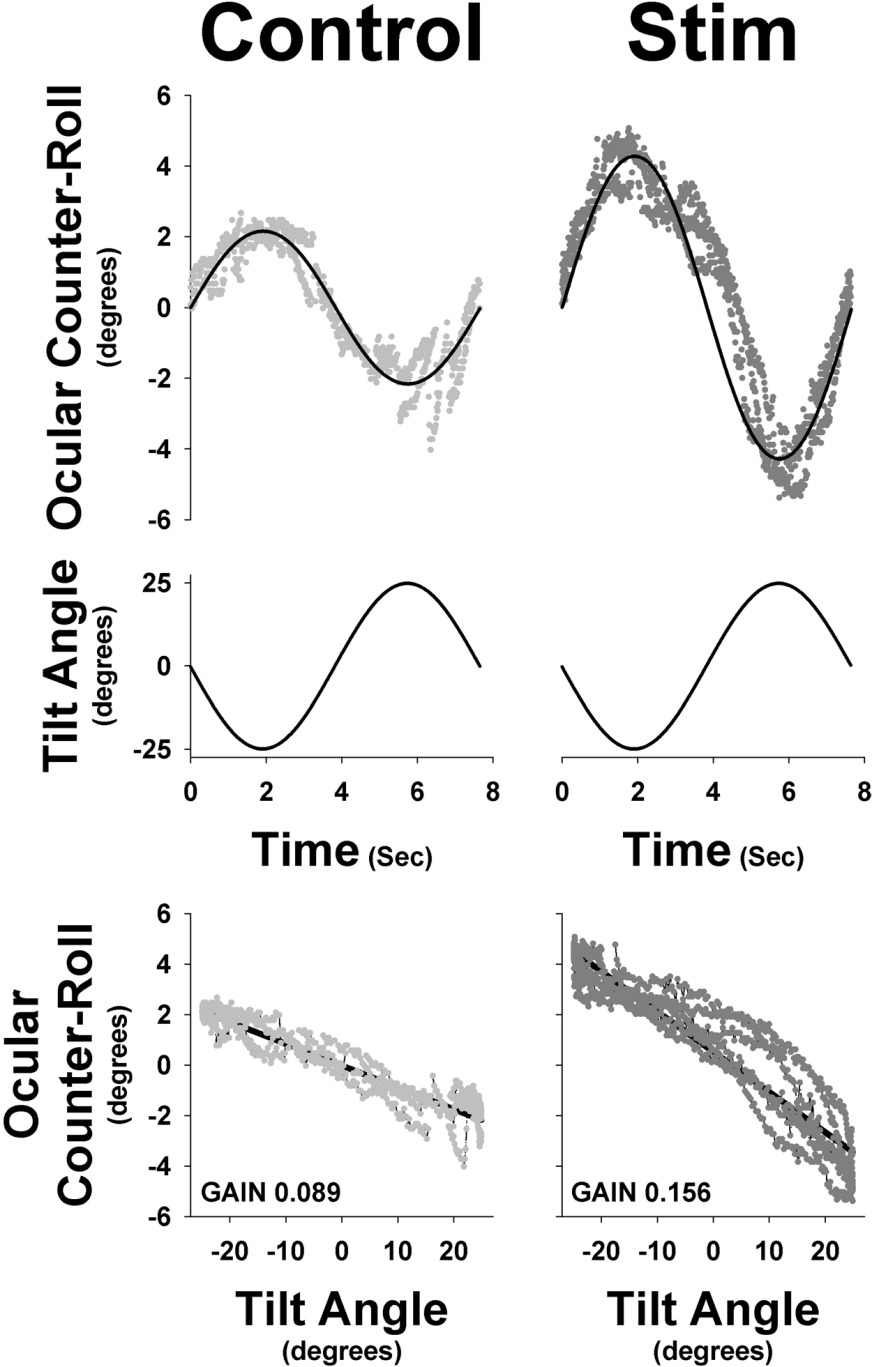
OCR of one subject to 0.125 Hz during ±25 deg passive roll tilt during both no stimulation and SN trials. The bottom panel demonstrates the gain calculations from the linear regression. This subject demonstrated a significant increase in OCR from control (left side) to stimulation (right side).

**Figure 2:**
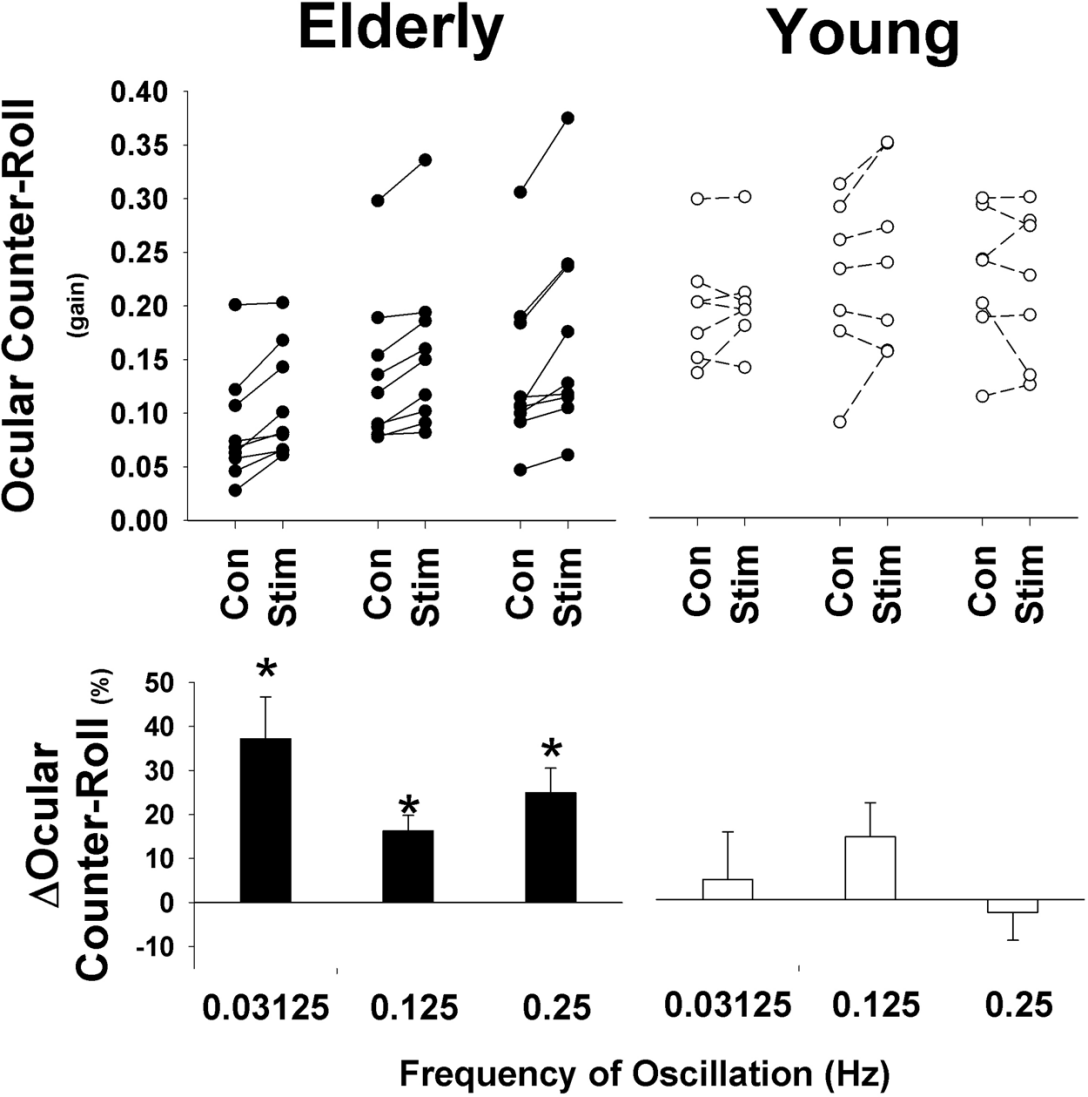
Change in OCR for Young and Elderly subjects from control to stim trials. The top panel shows individual data at each of the three stimulus frequencies, while the bottom panel shows the mean (±SEM). Elderly subjects demonstrated a significant increase in OCR at all motion frequencies suggesting improved vestibular function. Young subjects showed no significant change at any frequency.

Use of imperceptible stochastic noise stimulation resulted in significant increases in OCR gain in elderly individuals. Figure 1 shows OCR from one elderly subject at baseline and with stimulation during a roll frequency of 0.125 Hz. Electrical SN stimulation significantly increased OCR gain at all frequencies in all elderly subjects (P = 0.007) while producing no significant change in OCR gain in young subjects (Fig. 2). Table 1 depicts mean OCR gain values for young and elderly participants at each of the roll frequencies tested both with and without stimulation.

**Figure 3:**
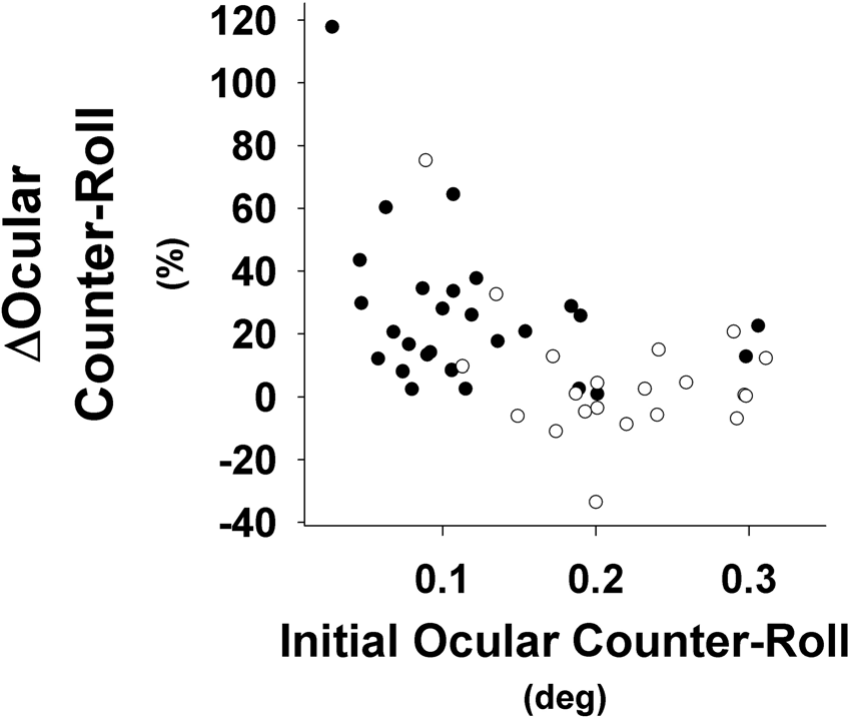
Relationship between baseline OCR and improvement (positive change) during electrical SN stimulation. Note that subjects with lower baseline values showed greatest improvement. Filled circles represent elderly individuals. Open circles represent younger participants.

Interestingly, subjects with the lowest baseline OCR gain demonstrated the greatest increases in OCR gain with stimulation (Fig. 3). This relationship was significant using both linear (R^2^ = 0.26, P = 0.003) and second-order quadratic functions (R^2^ = 0.40, P < 0.001). There was a significant interaction between age group, tilt frequency and stimulus condition (p = 0.018). When examining the raw data in Fig. 3, there appears to be a ceiling effect in that providing electrical SN in subjects with normal gain has limited or no effect. Thus, electrical SN appears to improve function in those with impaired otolith responses without inducing hypersensitivity or other adverse effects in those with normal function.

## Discussion

This study had two main findings. First, we observed an age-related loss in vestibular (otolith-ocular) function when comparing OCR gain in elderly subjects compared to a young subject group. This is consistent with previous studies that have shown age-related loss in otolith-ocular reflexes^5,6^. Second, we observed that imperceptible electrical SN significantly enhanced OCR gain in the elderly group. Since the increase in OCR was inversely proportional to baseline OCR gain, this type of an intervention should be generalizable to other patient populations with reduced otolith-ocular function^13^.

To our knowledge, this is the first study that has shown a positive effect of electrical SN on a gaze stabilization measure in subjects with reduced otolith-ocular function. While high levels of galvanic stimulation can elicit ocular torsion^11^, our thresholding technique ensured that our stimulus amplitudes did not provide a confounding additive effect but more likely enhanced the sensory transduction processing of tilt orientation. Our results are consistent with a recent study by Iwasaki *et al*.^16^ that demonstrated noisy galvanic stimulation can increase the amplitude of ocular VEMPs in healthy subjects. Ocular VEMPs assess the utricular-ocular reflex pathways by stimulating the peripheral end organs and measuring the ocular muscle activity^17^. Given that galvanic vestibular stimulation can mediate both otolith and canal responses^18^, the extent to which the improvement we observed was otolith mediated or both canal and otolith mediated is a topic for future research.

Our study was limited to subjects who were healthy with no recent neurological dysfunction or history of falls. While we observed reduced otolith-ocular function in the elderly group, a further extension of this work would be to test electrical SN patients with vestibular hypofunction as established using a larger battery of tests. This would be a critical next step to extend this proof of concept toward clinical applications. We observed a significant improvement in OCR in our elderly group using one level of electrical SN (90% of threshold). Another extension of our study would be to use graded intensities to further characterize how different SN levels would affect the OCR.

Recent evidence suggests that this type of peripheral stimulation influences both hair cells and vestibular afferent fibers^19^. Irregular neurons may be preferentially activated at the lower currents used with our intervention^20^. While the underlying mechanisms of how electrical SN enhances vestibular function remain unclear, one possibility is that addition of electrical noise causes fluctuations in membrane potentials, thus occasionally reducing depolarization necessary for neurons to fire. We would expect that this stimulation would improve tilt thresholds as well as other functions mediated by the vestibular system. Electrical SN has indeed been recently shown to improve other vestibular reflexes, such as posture^21,22^ and locomotion^23^.

Vestibular rehabilitation has shown efficacy in the treatment of the elderly^24^. Electrical SN may provide an enhancement to existing rehabilitation strategies. One key advantage of this type of non-invasive intervention is that it is both well tolerated by the elderly and does not increase cognitive load by requiring re-training. In addition, since the stimulation is noise based, neural systems theoretically will not adapt to the stimulus and thus long-term treatment should be more feasible. This study demonstrates that transcutaneous application of imperceptible stochastic noise stimulation can be used to enhance reduced otolith-ocular function in an elderly population.

## Methods

### Participants

The effects of aging in this study were inferred by comparing two subject groups. The young group consisted of seven subjects (four females, three males) with a mean age of 26 yrs (range 21–39 yrs). The older group consisted of nine subjects (seven females, two males) with a mean age of 71 yrs (range of 62–82 yrs). Based on a brief medical history questionnaire, all subjects were healthy with no recent neurological dysfunction or history of falls. This study protocol was approved by the Beth Israel Deaconess Medical Centre Institutional Review Board. Informed consent was obtained from each subject prior to participation. All experiments were performed in accordance with relevant guidelines and regulations.

### Measurement of OCR

OCR measurements were performed using a procedure we have previously published^5^. In summary, subjects were passively roll-tilted in darkness while fixating a small LED target at 1.4 m. This far target distance has been more sensitive to age-related OCR loss than near targets^6^. Subjects were tilted about their naso-occipital axis at eye level, with the head and torso stabilized using adjustable straps and padding to move en bloc with the chair. Each subject was tilted ±25 degrees at three frequencies: 0.03125 Hz (3.125 degrees/second), 0.125 Hz (12.5 degrees/second) and 0.2 Hz (20 degrees/second) for both control and stochastic noise trials (Fig. 2). Previous work has demonstrated that ocular reflexes in the 0.03125 Hz frequency are primarily otolith mediated while higher frequencies involve increasing canal inputs^8^. Stimulus conditions were random and counterbalanced across subjects.

Torsional, horizontal and vertical eye positions were derived from a near-infrared 3D videography system using custom software to allow recording in darkness^25^. Changes in horizontal and vertical eye position and pupil radius were negligible due to constant fixation on the wall target. Changes in torsional eye movements derived from natural landmarks in the iris represented the OCR response during roll tilt (Fig. 1). OCR gain was calculated as ratio of deg of torsion to tilt using least-squared regression. Effects of stochastic noise on OCR were expressed as a percentage change from baseline.

### Electrical stimulation

**Figure 4:**
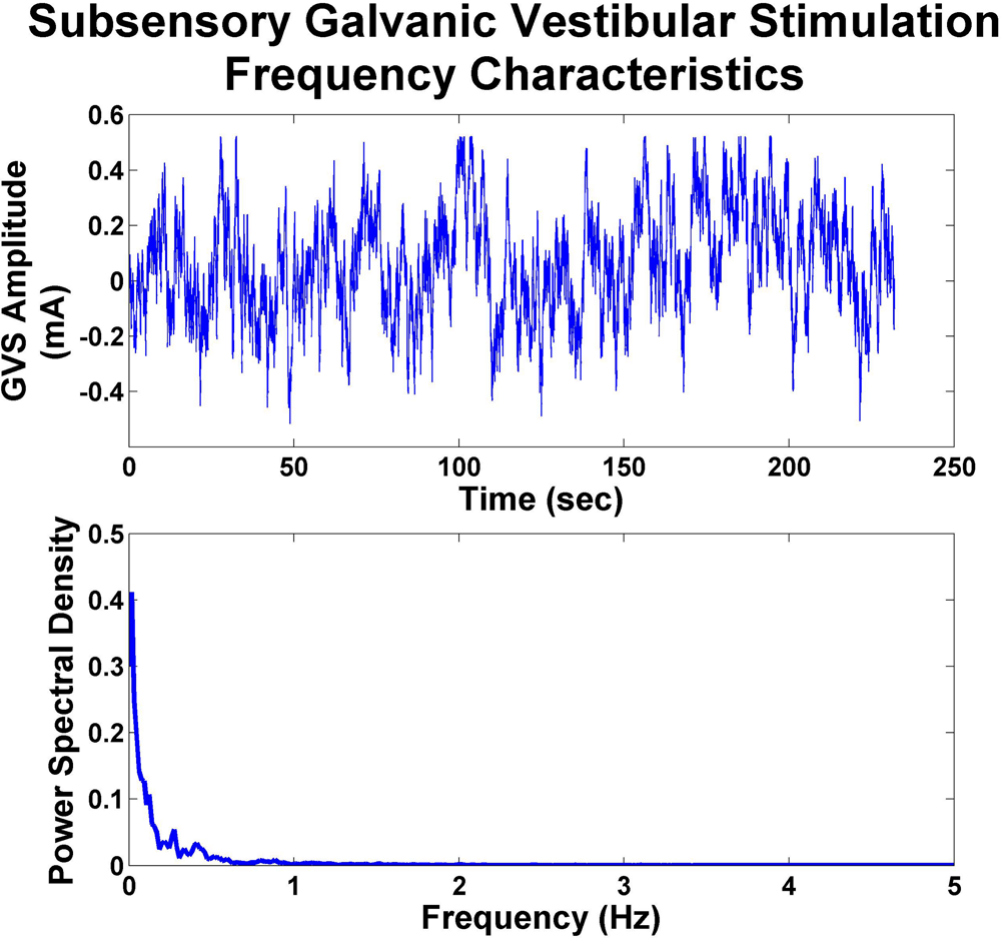
Sample of the stochastic noise signal over time for one subject (top graph) with the corresponding power spectral density curve for the same signal (bottom graph). Note that while the stimulus amplitude was customized for each subject (see text), the frequency bandwidth was maintained consistently across subjects so that the stimulus was predominantly banded in the low frequency range (95% < 2 Hz).

Stochastic noise signals were generated using Labview (National Instruments, Texas, USA) and applied bilaterally through a current isolator to large surface electrodes (5 × 5 cm) located on each mastoid process. The electrode site was cleaned with alcohol and skin abrasive to ensure low impedance to minimize pinprick tactile cues. The spectrum of stochastic noise was generated using an equation modified from that described by Collins *et al*.^10^ where the majority of the power of the signal falls below 2 Hz (Fig. 4). Stimulus amplitude was set at 90% of a threshold value determined for each subject by applying a constant stimulus starting at 0 mA and increasing in 0.1 mA increments. The threshold was the lowest stimulus value at which either the subjects reported an awareness of the galvanic stimulus (8 of 16 subjects) or we observed the onset of at least 3 consecutive nystagmus beats in a consistent direction (the remaining 8 subjects). To ensure input noise was sub-perceptual threshold, we confirmed that subjects reported no awareness of stimulus during the SN trials. Stimulus amplitudes were 1.6 ± 0.9 mA for elderly and 1.1 ± 0.5 mA for younger subjects.

### Statistical Analysis

All statistical tests were performed using SPSS 24 using a GLM repeated measures analysis with age as a between-subjects factor (Young vs Elderly) and frequency (0.03125 vs 0.125 vs 0.2 Hz) and stochastic noise (Sham vs Stim) as within-subjects repeated measures. This mixed-model multivariate ANOVA used Wilks’ lambda as the critical statistic with an alpha significance level of 0.05. Post-hoc analyses to compare stimulus frequencies used Bonferroni correction to reduce Type I error. Least squares linear and quadratic fits were used to evaluate the effect of initial OCR on percent change between sham and stim, with coefficient of determination as the key outcome measure. All data are described using SEM to facilitate comparison of means.

### Data availability

The datasets generated during and/or analyzed during the current study are available from the corresponding author on reasonable request.
